# Supplementary material for: Clonal analysis and dynamic imaging identify multipotency of individual Gallus gallus caudal hindbrain neural crest cells toward cardiac and enteric fates
Source: Nat Commun. 2021 Mar 25;12:1894. doi: 10.1038/s41467-021-22146-8 (PMC7994390; doi:10.1038/s41467-021-22146-8)
Supplement: Supplementary file 1 — Supplementary Information [file 41467_2021_22146_MOESM1_ESM.pdf]

Clonal analysis and dynamic imaging identify multipotency of individual *Gallus gallus* caudal hindbrain neural crest cells toward cardiac and enteric fates

Supplementary Information

Weiyi Tang[1], Yuwei Li[1], Ang Li[2] and Marianne E. Bronner[1]*

[1]Division of Biology and Biological Engineering, California Institute of Technology, Pasadena, CA 91125

[2]Department of Kinesiology, College of Nursing and Health Innovation, University of Texas at Arlington, Arlington, TX 76019

*Corresponding to: mbronner@caltech.edu

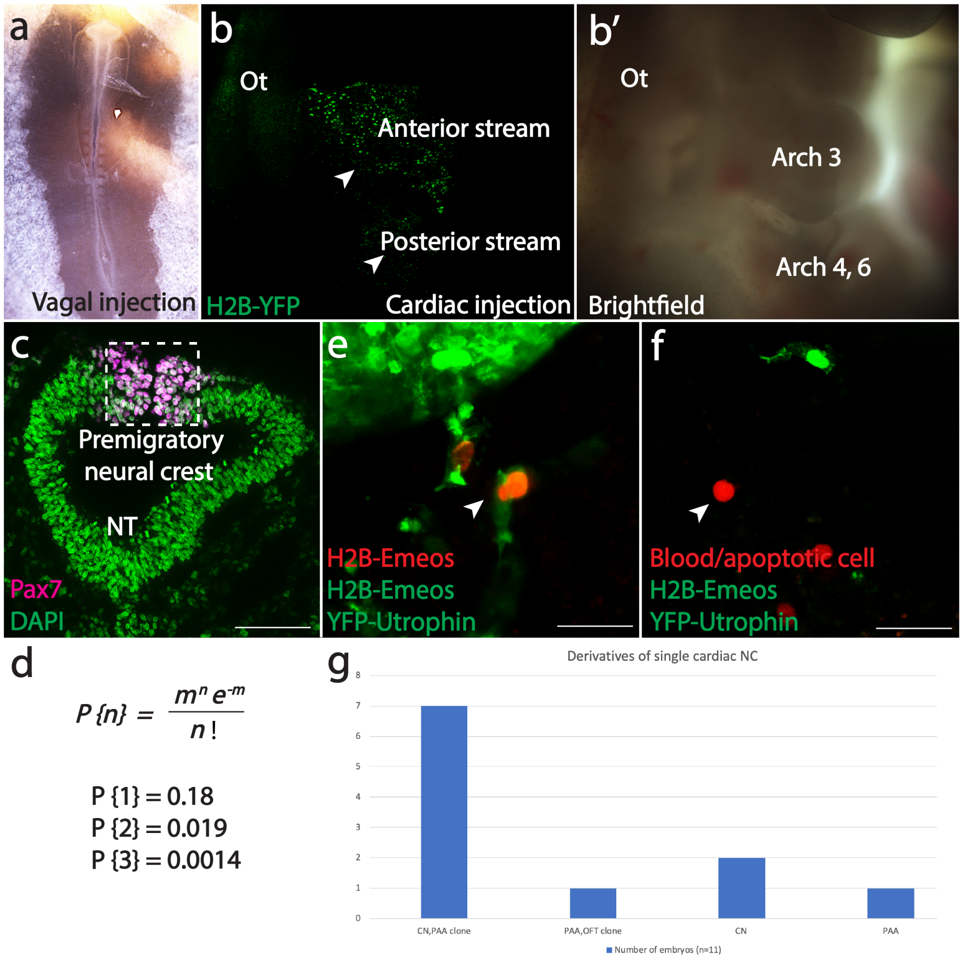

Supplementary Figure 1: Supplementary information for clonal analysis and cardiac neural crest single-cell photoconversion. (a) A chick embryo immediately after injection. The entire region of the neural tube adjacent to mid-otic to somite 7 is labelled with viral mixture (blue). Note that the first two somites are disappearing. **(b, b')** An embryo was specifically injected at the cardiac level between mid-otic to somite 3. 48 hours post injection, virally labelled cells (green) migrated exclusively into pharyngeal arches 3, 4, 6, suggesting axial specificity (23/23). **(c)** To estimate probability of double and triple infections, number of neural crest precursor cells (Pax7+, magenta) and total cells in the hindbrain (DAPI, green) were quantified (12/12). Amongst all viral particles injected, particles infecting Pax7+ cells can be derived by the ratio between Pax7+ cells and total cells in the hindbrain (~0.258). **(d)** At the vagal level, approximately 1292 viral particles infected ~5863 Pax7+ cells with equal probability. Following a Poisson distribution of multiple infection[21], probability of double infection and triple infection is 0.019 and 0.0014

for vagal neural crest cells, respectively (n, number of viral particle(s) infecting a cell; m, ratio of viral particle number and Pax7+ cell number). **(e)** 12 hours post-conversion, the converted cell divided into two daughter cells, with red H2B-Emeos in the nucleus and YFP-Utrophin delineating cellular structures. **(f)** Although blood cells or apoptotic cells can also possess red nuclear-like signal, YFP-Utrophin will not be present in those cells. Thus, the cell in f (arrowhead) was not counted as a derivative of the converted cell. **(g)** Number of bi-location and uni-location cardiac clones observed in single-cell photoconversion assay. Abbreviations: CN, cranial nerve; PAA, pharyngeal arch arteries; OFT, outflow tract. Numbers in parenthesis shows the number of images with the phenotype presented in the figure out of all images taken.

Scale bars: c 160μm; e, f 20μm.

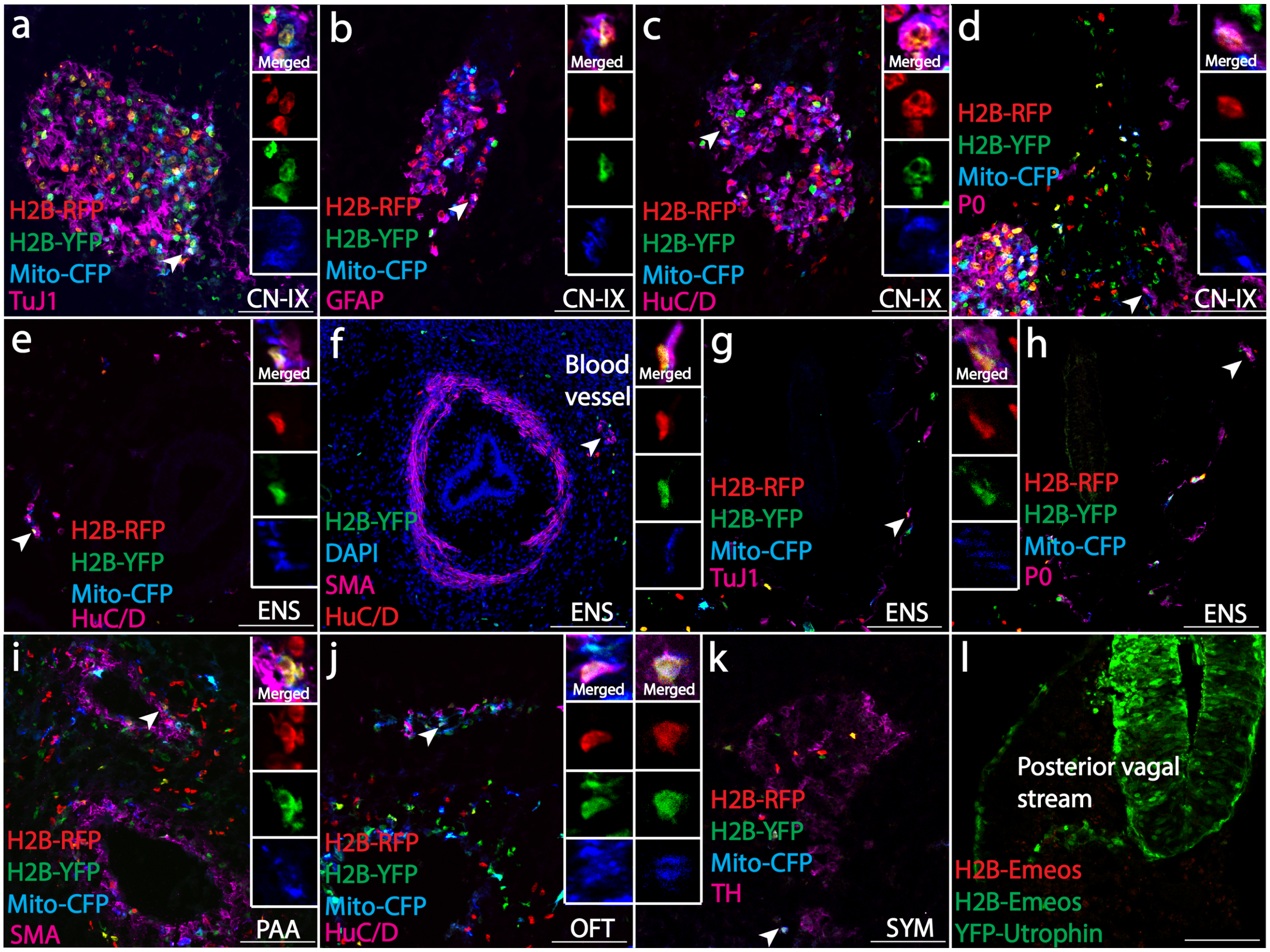

Supplementary Figure 2: Cell fate analysis of clonally labelled vagal neural crest cells and stream-specific photoconversion. At E7, in H2B-RFP, H2B-YFP, Mito-CFP triple infected clone, cells acquired diverse cell fates, including neurons **(a, c)**, glia **(b)** and Schwann cells **(d)** in cranial nerve nine (CN-IX) and surrounding tissue. In the enteric nervous system (ENS), they gave rise to neuronal **(e, g)** and Schwann cells **(h)**. However, neural crest cells did not generate smooth muscle layer of the gut, despite in blood vessels (arrowhead) **(f)**. Cells of the clone also contributed to smooth muscle surrounding pharyngeal arch arteries **(i)**, neuronal cells in the outflow tract **(j)**, and in rare cases posterior sympathetic neurons (SYM) **(k)**. **(l)** In stream-specific photoconversion, a transverse section at posterior vagal region was obtained. The ventral portion is presented in Fig3r. In the dorsal portion, no photoconverted cell was visible, indicating that photoconversion was specific for cardiac neural crest cells.

Scale bars: a-k 100μm; l 160μm.

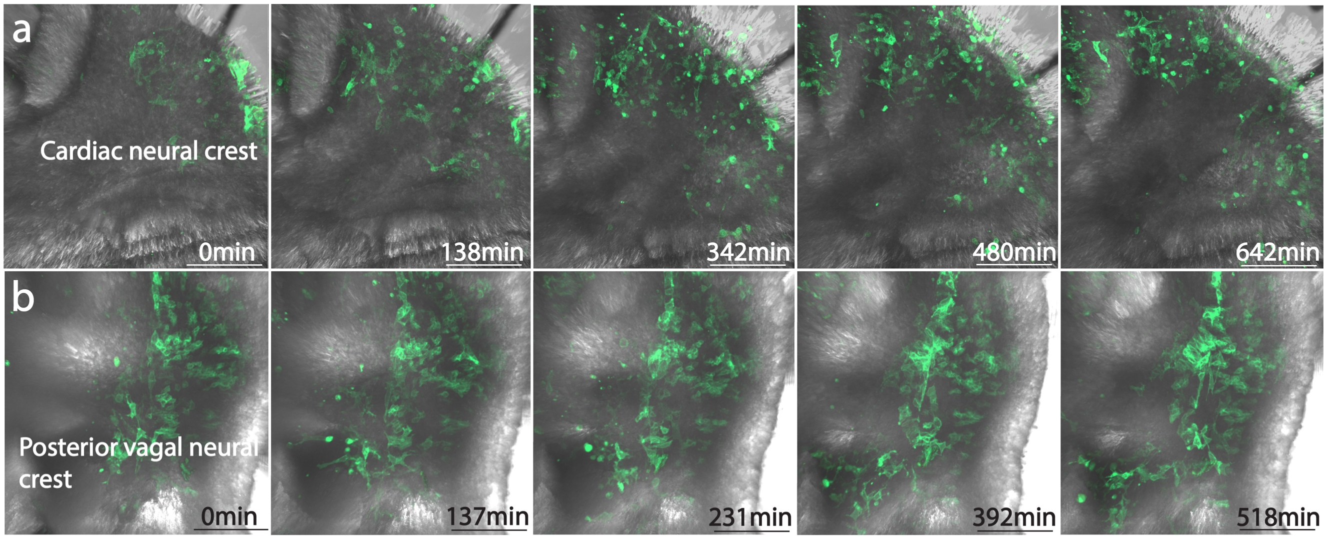

Supplementary Figure 3: Individual cell migration of vagal neural crest cells. Time-lapse imaging with the brightfield (grey) view on the cardiac **(a)** and posterior vagal **(b)** neural crest cells expressing membrane-YFP.

Scale bars: a, b 100μm.

Supplementary Table 1: Primer list.

Primer name	Construct	Sequence (5'-3')
H2B_Not1_for	RIA-H2B-CFP	taagcagcggccgcatgccagagccagcgaagtct
CFP_Cla1_rev	RIA-H2B-CFP	taagcaatcgatttacttgtacagctcgtccatgccga
H2B_Asc1_for	RIA-H2BEmeos-T2A-YFPUtrophin	caggcgcgccatgccagagccagcgaagtct
Emeos_Spe1_rev	RIA-H2BEmeos-T2A-YFPUtrophin	gcactagtggatcctcgtctggcattgtc
YFP_ Not1_for	RIA-H2BEmeos-T2A-YFPUtrophin	gcgcggccgcatggtgagcaagggcgaggag
Utrophin_Cla1_rev	RIA-H2BEmeos-T2A-YFPUtrophin	gaatcgatttagtctatggtgacttgctgag
DNFgfr1_Not1_for	RIA- H2BEmeos -T2A-DNFgfr1	taagcagcggccgcatgtttacctggaggtgcctcatcctt
DNFgfr1_Cla1_rev	RIA- H2BEmeos -T2A-DNFgfr1	taagcaatcgatttatgttacctgtctgcgcagtgggatg
DNCxcr4_Not1_for	RIA- H2BEmeos -T2A-DNCxcr4	taagcagcggccgcatgtccattcctttgcctcttttgcagatatacac
DNCxcr4_Cla1_rev	RIA- H2BEmeos -T2A-DNCxcr4	taagcaatcgatttatttaaatttggctccaaggaaagcatagaggatgg
DNRet_Acs1_for	RIA- DNRet -T2A-H2BEmeos	taagcaggcgcgccatggtgcccttcccggtgac
DNRet_Spe1_rev	RIA- DNRet -T2A-H2BEmeos	taagcaactagtcctctccatccggtggccgg
H2B_Not1_for	RIA- DNRet -T2A-H2BEmeos	taagcagcggccgcatgccagagccagcgaagtct
Emeos_Cla1_rev	RIA- DNRet -T2A-H2BEmeos	taagcaatcgatttaggatcctcgtctggcattgtcagg
