# Supplementary material for: Clonal analysis and dynamic imaging identify multipotency of individual Gallus gallus caudal hindbrain neural crest cells toward cardiac and enteric fates
Source: Nat Commun. 2021 Mar 25;12:1894. doi: 10.1038/s41467-021-22146-8 (PMC7994390; doi:10.1038/s41467-021-22146-8)
Supplement: Supplementary file 2 — Reporting Summary [file 41467_2021_22146_MOESM2_ESM.pdf]

nature research

Corresponding author(s): Marianne E. Bronner

Last updated by author(s): Feb 10, 2021

Reporting Summary

Nature Research wishes to improve the reproducibility of the work that we publish. This form provides structure for consistency and transparency in reporting. For further information on Nature Research policies, see Authors & Referees and the Editorial Policy Checklist .

Statistics

For all statistical analyses, confirm that the following items are present in the figure legend, table legend, main text, or Methods section.

n/a	Confirmed	
☐	☒	The exact sample size (*n*) for each experimental group/condition, given as a discrete number and unit of measurement
☐	☒	A statement on whether measurements were taken from distinct samples or whether the same sample was measured repeatedly
☐	☒	The statistical test(s) used AND whether they are one- or two-sided<br>*Only common tests should be described solely by name; describe more complex techniques in the Methods section.*
☒	☐	A description of all covariates tested
☒	☐	A description of any assumptions or corrections, such as tests of normality and adjustment for multiple comparisons
☐	☒	A full description of the statistical parameters including central tendency (e.g. means) or other basic estimates (e.g. regression coefficient) AND variation (e.g. standard deviation) or associated estimates of uncertainty (e.g. confidence intervals)
☒	☐	For null hypothesis testing, the test statistic (e.g. *F*, *t*, *r*) with confidence intervals, effect sizes, degrees of freedom and *P* value noted<br>*Give P values as exact values whenever suitable.*
☒	☐	For Bayesian analysis, information on the choice of priors and Markov chain Monte Carlo settings
☒	☐	For hierarchical and complex designs, identification of the appropriate level for tests and full reporting of outcomes
☒	☐	Estimates of effect sizes (e.g. Cohen's *d*, Pearson's *r*), indicating how they were calculated

Our web collection on statistics for biologists contains articles on many of the points above.

Software and code

Policy information about availability of computer code

Data collection	Position information (x, y, z) in dynamic imaging was collected using IMARIS 9.1.0 and ZEN (blue edition); Images were processed and quantified using FIJI v1.0.
Data analysis	The squared displacement (SD) analysis was conducted using Mean square displacement analysis of particles trajectories package in MATLAB R2017a. Plots of initial coordinates against displacement distances were generated using MATLAB R2017a. Box-and-dot plots were generated using ggplot2 package in R. Wilcoxon rank-sum test was used for statistical comparison between two groups.

For manuscripts utilizing custom algorithms or software that are central to the research but not yet described in published literature, software must be made available to editors/reviewers. We strongly encourage code deposition in a community repository (e.g. GitHub). See the Nature Research guidelines for submitting code & software for further information.

Data

Policy information about availability of data

All manuscripts must include a data availability statement. This statement should provide the following information, where applicable:

- Accession codes, unique identifiers, or web links for publicly available datasets
- A list of figures that have associated raw data
- A description of any restrictions on data availability

All data generated and analyzed in this study are included in the article.

Field-specific reporting

Please select the one below that is the best fit for your research. If you are not sure, read the appropriate sections before making your selection.

Life sciences study design

All studies must disclose on these points even when the disclosure is negative.

Sample size	Numbers were determined based on sufficient consistencies between samples and the number of cells needed for position analysis in dynamic imaging.
Data exclusions	No data were excluded.
Replication	Biological replicates were used for all experiments. In multiplexed retroviral clonal analysis, slice culture was performed in 14, 12, or 9 replicates at cardiac, posterior vagal, or both regions, respectively; For clonal analysis via photoconversion, we performed single-cell photoconversion with n = 11 replicates on cardiac neural crest, n = 5 replicates on posterior vagal neural crest cell and n = 2 replicates on all post-otic migrating cardiac crest cells for stream-specific photoconversion. Immunohistochemistry for premigratory Pax7+ cells was performed in n=3 embryos.Molecular perturbation was performed with n = 11 replicates for RIA-DN-Fgfr11-YFP, n = 4 replicates for DN-Cxcr4-YFP or n = 10 replicates for DN-Ret-YFP (8/10 injected with H2B-RFP control). Time-lapse imaging for cardiac, posterior vagal and lateral view were performed successfully with 2, 1, 2 replicates. Replicate numbers for time-lapse imaging is relatively low since success rate was low due to tissue drifting during 8-10 hour sessions. To analyze cell trajectories, we tracked n= 21 and n = 18 individual cells for cardiac and posterior vagal migrating cells.
Randomization	In molecular perturbation of RET, H2B-RFP virus was used as control in the same embryo. Randomization was achieved by infection events.
Blinding	Blinding was not relevant to this study. Retroviral lineage analysis is non-invasive approach that label wild-type cells, thus comparison is not relevant. In molecular perturbation of RET, mutant and control virus infect cells in the same embryo so will be compared in the same context.

Reporting for specific materials, systems and methods

We require information from authors about some types of materials, experimental systems and methods used in many studies. Here, indicate whether each material, system or method listed is relevant to your study. If you are not sure if a list item applies to your research, read the appropriate section before selecting a response.

Materials & experimental systems

n/a	Involved in the study	
☐	☒	Antibodies
☐	☒	Eukaryotic cell lines
☒	☐	Palaeontology
☐	☒	Animals and other organisms
☒	☐	Human research participants
☒	☐	Clinical data

Methods

n/a	Involved in the study	
☒	☐	ChIP-seq
☒	☐	Flow cytometry
☒	☐	MRI-based neuroimaging

Antibodies

Antibodies used	Primary antibodies used: 1:500 Mouse anti-smooth muscle actin IgG2a, Sigma-Cat#A2547, clone 1A4 monoclonal; 1:500 Mouse anti-HuC/D IgG2b, Invitrogen-Cat#A21271, clone 16A11 monoclonal; 1:500 Mouse anti-TuJ1 IgG2a, Biolegend-Cat# 801201, clone TUJ1 monoclonal; 1:500 Rabbit anti-Tyrosine Hydroxylase (TH), Millipore-Cat# AB152, polyclonal; 1:10 8-IE7 (monoclonal mouse anti-GFAP IgG1), DSHB, 1:10 3H5 (monoclonal mouse anti-HNK1 IgM), DSHB, 1:10 PAX7 (monoclonal mouse anti-Pax7 IgG1), DSHB, 1:10 IE8 (monoclonal mouse anti-P0 IgG1), DSHB.<br>Secondary antibodies used: 1:1000 goat anti-mouse IgG2a 633, #A21136 (polyclonal), 1:1000 goat anti-mouse IgG1 647, #A21240 (polyclonal), 1:1000 goat anti-mouse IgG2b 647, #A21146 (polyclonal), 1:1000 goat anti-mouse IgM 647, #A21238 (polyclonal), 1:1000 donkey anti-rabbit 647, #A31573 (polyclonal). All secondary antibodies from Invitrogen Molecular Probes).
Validation	Validations are available on DSHB website for GFAP, HNK1, Pax7, P0 antibodies; Sigma website for Mouse anti-smooth muscle actin, Invitrogen for mouse antiHuC/D, Biolegend for Mouse anti-TuJ1, Millipor for Rabbit anti-Tyrosine Hydroxylase.<br>GFAP: species reactivity Mouse, Rat; applications include ELISA, Immunohistochemistry, Western Blot.<br>HNK1: species reactivity Chicken, Human, Lamprey, Shark; applications include Immunofluorescence, Immunohistochemistry,<br>Pax7: species reactivity Amphibian, Avian, Bovine, Canine, Fish, Goat, Human, Mouse, Ovine, Porcine, Quail, Rat, Turtle, Xenopus, Zebrafish; applications include Chromatin Immunoprecipitation, FACS, FFPE, Gel Supershift, Immunofluorescence, Immunohistochemistry, Immunoprecipitation, Western Blot.<br>P0: species reactivity Chicken, Planaria, Quail; applications include Immunohistochemistry, Western Blot.<br>Mouse anti-smooth muscle actin: species reactivity human, frog, sheep, chicken, goat, bovine, rat, guinea pig, mouse, canine, rabbit, snake; applications include immunohistochemistry, indirect immunofluorescence, western blot.<br>mouse antiHuC/D: species reactivity for Avian, Chicken, Human, Zebrafish; applications include immunohistochemistry, immunofluorescence, western blot, ELISA, immunocytochemistry.<br>Mouse anti-TuJ1: species reactivity Human, Mouse, Rat; verified applications include immunohistochemistry, western blot.<br>Rabbit anti-Tyrosine Hydroxylase: species reactivity Human, Mouse, Rat, applications include ELISA, immunocytochemistry,

	immunohistochemistry, immunofluorescence, western blot.

Eukaryotic cell lines

Policy information about cell lines

Cell line source(s)	Chick DF1 cells (ATCC, Manassas, VA; #CRL-12203, Lot number 62712171).
Authentication	Certificate of Analysis through COI assay (interspecies) available at ATCC website.
Mycoplasma contamination	Certificate of Analysis with negative mycoplasma testing through Hoechst DNA stain, agar culture, PCR-based assay available at ATCC website.
Commonly misidentified lines (See ICLAC register)	No commonly misidentified lines was used.

Animals and other organisms

Policy information about studies involving animals; ARRIVE guidelines recommended for reporting animal research

Laboratory animals	Laboratory animals involve E1-E7 chick (Gallus gallus) embryos. Sex is randomized, as embryos were not pre-selected based on sex.
Wild animals	No wild animal was involved.
Field-collected samples	No field collected sample was involved.
Ethics oversight	None. This project uses chick embryos from E1-E7, which are not considered vertebrate animals until E10.

Note that full information on the approval of the study protocol must also be provided in the manuscript.
