# Supplementary material for: Clonal analysis and dynamic imaging identify multipotency of individual Gallus gallus caudal hindbrain neural crest cells toward cardiac and enteric fates
Source: Nat Commun. 2021 Mar 25;12:1894. doi: 10.1038/s41467-021-22146-8 (PMC7994390; doi:10.1038/s41467-021-22146-8)
Supplement: Supplementary file 9 — Description of Additional Supplementary Files [file 41467_2021_22146_MOESM9_ESM.pdf]

Title: Supplementary Data 1:
Description: Number of cells in clonal analysis on the (a) cardiac, (b) posterior vagal, (c) vagal levels. Abbreviations: dNT, dorsal neural tube; CN, cranial nerve nine; PAA, pharyngeal arch arteries; OFT, outflow tract; DRG, dorsal root ganglia; SYM, sympathetic chain; ENS, enteric nervous system. Average group clone size: the average size of clones with a specific number of derivatives. Average clone size: average size of all clones observed.

Title: Supplementary Data 2:
Description: Quantitative analysis of neural crest cell migration under *CXCR4* and *RET* signaling perturbation. To analyze the role of *CXCR4* in cardiac neural crest migration into the enteric nervous system, images of the foregut and cranial nerve nine were quantified. Average cell number in the foregut was normalized to cell number in cranial nerve nine, to which neural crest contribution is not affected by *CXCR4*. The normalized values were similar between DN-Cxcr4 and H2B-YFP (control) virus injected embryos, indicating that cell migration to the enteric nervous system is not affected by *CXCR4* perturbation. To investigate the role of *RET* signaling during cardiac neural crest migration into the foregut, number of DN-Ret expressing and H2B-RFP (control) expressing cells in the foregut was normalized to their counterparts in the pharyngeal junction (anterior to the foregut) to account for titer variations. Loss of *RET* signaling led to ~86% reduction of cardiac neural crest cells migrating to the foregut.

Title: Supplementary Movie 1:
Description: Cardiac neural crest migration, corresponding to Fig4a.

Title: Supplementary Movie 2:
Description: Posterior vagal neural crest migration, corresponding to Fig4g.

Title: Supplementary Movie 3:
Description: Lateral view of cardiac neural crest migration along anterior-posterior axis (express Utrophin-Scarlet), corresponding to Fig4n.

Title: Supplementary Movie 4:
Description: Lateral view of cardiac neural crest migration along anterior-posterior axis, replicate (express H2B-YFP), corresponding to Fig4n’.
